# Controlled Release of rAAV Vectors from APMA-Functionalized Contact Lenses for Corneal Gene Therapy

**DOI:** 10.3390/pharmaceutics12040335

**Published:** 2020-04-09

**Authors:** Fernando Alvarez-Rivera, Ana Rey-Rico, Jagadeesh K Venkatesan, Luis Diaz-Gomez, Magali Cucchiarini, Angel Concheiro, Carmen Alvarez-Lorenzo

**Affiliations:** 1 Departamento de Farmacología, Farmacia y Tecnología Farmacéutica, I+DFarma, Facultad de Farmacia and Health Research Institute of Santiago de Compostela (IDIS), Universidade de Santiago de Compostela, 15782 Santiago de Compostela, Spain; fer.alvarez.rivera@gmail.com (F.A.-R.); luis.diaz.gomez@usc.es (L.D.-G.); angel.concheiro@usc.es (A.C.); 2 Cell Therapy and Regenerative Medicine Unit, Centro de Investigacións Científicas Avanzadas (CICA), Universidade da Coruña, Campus de A Coruña, 15071 A Coruña, Spain; ana.rey.rico@udc.es; 3 Center of Experimental Orthopaedics, Saarland University Medical Center, 66421 Homburg, Germany; jegadish.venki@gmail.com (J.K.V.); mmcucchiarini@hotmail.com (M.C.)

**Keywords:** therapeutic contact lens, gene therapy, rAAV vectors, controlled release, corneal diseases

## Abstract

As an alternative to eye drops and ocular injections for gene therapy, the aim of this work was to design for the first time hydrogel contact lenses that can act as platforms for the controlled delivery of viral vectors (recombinant adeno-associated virus, rAAV) to the eye in an effective way with improved patient compliance. Hydrogels of hydroxyethyl methacrylate (HEMA) with aminopropyl methacrylamide (APMA) (H_1_: 40, and H_2_: 80 mM) or without (H_c_: 0 mM) were synthesized, sterilized by steam heat (121 °C, 20 min), and then tested for gene therapy using rAAV vectors to deliver the genes to the cornea. The hydrogels showed adequate light transparency, oxygen permeability, and swelling for use as contact lenses. Loading of viral vectors (rAAV-*lacZ*, rAAV-RFP, or rAAV-hIGF-I) was carried out at 4 °C to maintain viral vector titer. Release in culture medium was monitored by fluorescence with Cy3-rAAV-*lacZ* and AAV Titration ELISA. Transduction efficacy was tested through reporter genes *lacZ* and RFP in human bone marrow derived mesenchymal stem cells (hMSCs). *lacZ* was detected with X-Gal staining and quantified with Beta-Glo^®^, and RFP was monitored by fluorescence. The ability of rAAV-hIGF-I-loaded hydrogels to trigger cell proliferation in hMSCs was evaluated by immunohistochemistry. Finally, the ability of rAAV-*lacZ*-loaded hydrogels to transduce bovine cornea was confirmed through detection with X-Gal staining of β-galactosidase expressed within the tissue.

## 1. Introduction

Ocular gene therapy has the potential to cure or relieve symptoms of inherited or acquired diseases by replacing a defective gene with a normal gene [1,2]. This chance to cure by correcting the cause of the disorder means an important advantage compared with conventional drugs that, in most cases, only can suppress symptoms [3,4]. Furthermore, gene therapy provides long-term benefits compared with small drugs for ocular therapeutics whose effects usually last few hours. Although there are numerous clinical trials on-going involving a variety of genes and carriers [5], the recent approval of Voretigene (Luxturna) for the treatment of inherited retinal dystrophy, which can repair one abnormal gene halting retinal degeneration and even restoring sight, has represented the clinical launching of ocular gene therapy [6].

Besides retina, cornea represents an attractive target for gene therapy because of its accessibility, immune privilege, stability in vivo, and transparency; factors that facilitate gene delivery and monitoring for corneal diseases (e.g., corneal haze, corneal neovascularization, herpetic stromal keratitis, mucopolysaccharidosis VII, corneal transplantation, and graft rejection, among others) [7,8,9,10]. Cornea is an avascular tissue that refracts the light (together with the lens) and acts as a first protective barrier due to the tight junctions of the epithelium and Bowman’s membrane. This important barrier function along with other eye protective mechanisms (e.g., lachrymal fluid replacement, blinking, nasolachrymal drainage, etc.) represents a challenge to achieve sufficient drug bioavailability in the ocular structures after topical application of eye drops [9,11].

Several approaches have been tested to drive genes to the cornea [1,4,7,12]. Non-viral vector therapy relies on physical methods (mechanical, electrical, or surgical procedures) that deliver naked DNA, or chemical methods (high salt solutions and polycation carriers) that enhance entry of nucleic acid into cells [1,10,12,13,14]. Naked DNA is quite unstable, and thus a variety of non-viral nanocarriers based on inorganic particles, liposomes, and cationic lipids and polymers (e.g., polylysine, polyarginine, chitosan) have been proposed [4,15]. Typically, non-viral vectors may establish electrostatic interactions with both the nucleic acid (forming complexes) and the cell surface facilitating attachment and subsequent endocytosis [16]. Differently, viral vectors encapsulate DNA and develop a strong transduction activity, being so far the gene delivery system of choice for ocular diseases [5,16,17]. Adenoviral vectors (AV), recombinant adeno-associated viral vectors (rAAV), retroviral vectors (RV), and lentiviral vectors (LV) are the most common in gene therapy, although other viral vectors such as herpes simplex virus or baculovirus have also been tested. Viral vector selection depends on the target cell and the required effect duration (i.e., long- or short-term response). In this regard, rAAV are particularly attractive because of their small size, high transduction efficiency, and safety [18,19,20], as demonstrated for Voretigene (Luxturna) [6] and also in a wide variety of different applications [17,18]. The adeno-associated virus serotype 2 vector (rAAV-2) is one of the most suitable to transduce corneal tissues [6,10,21,22], although other serotypes (i.e., rAAV-6, rAAV-8 and rAAV-9) have also been tested [23,24].

The administration route of gene delivery systems for ocular treatment is also an important factor to take into account. Some studies have focused on systemic administration in neonate animal models, but retinal–blood barrier impairs gene transduction in adults [25,26]. Nasal administration has been explored for vaccination purposes using DNA encoding TGF-β to suppress immunity and modulate the immunoinflamatory response to herpes simplex virus (HSV) eye infection [27]. Direct ocular administration using invasive methods (intrastromal, intralimbal, subconjunctival, intracameral, and intravitreal injections) [5,6,12] is still the most usual mode of administration since it allows reaching substantial gene expression. Nevertheless, repeated applications of subconjunctival or intravitreal injections are associated with vitreous hemorrhages, endophthalmitis, and cataract [26].

**Figure 1:**
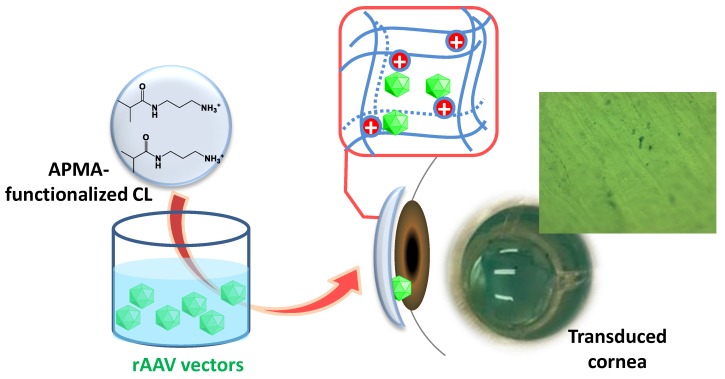
Hydrogel contact lenses may be functionalized to host viral vectors and to provide their sustained delivery, and thus transfection, to the ocular structures.

Contact lenses (CLs) attract great interest as drug delivery platforms able to provide sustained levels to the post-lens lachrymal fluid, i.e., the volume confined between the CL and the cornea. Attenuated turnover of the post-lens lachrymal fluid facilitates the reaching of higher drug concentrations that prompt the diffusion through the cornea, while non-productive absorption is minimized [28,29,30,31]. Safety of CLs for prolonged wearing to correct vision suggests that they can be considered as patient-friendly drug delivery devices. However, ocular gene therapy approaches using CLs have not been evaluated so far. It can be hypothesized that sustained release from CLs of nonreplicating rAAV capsids to the post-lens lachrymal fluid may be useful for the transduction of the epithelium layer, avoiding rapid dilution, neutralization by antibodies, or clearance by the lachrymal fluid [32] (Figure 1). Although hydrogels have been tested as platforms for non-viral vectors delivery [33], to the best of our knowledge this is the first attempt to use hydrogels as controlled delivery systems of viral vectors. Charged viral vectors such as rAAV, AV, and LV [34] could interact through electrostatic bonds with ionic CLs. Thus, the aim of this work was to provide a proof-of-principle of the suitability of functionalized poly(2-hydroxyethyl methacrylate) (p-HEMA) hydrogels as platforms that host and release rAAV vectors for sustained gene therapy of corneal complications, while keeping adequate mechanical and optical properties to act as ocular bandages or CLs. These hydrogels should allow clear vision or even correct refractive errors at the same time. Since rAAV capsids have a pI in the range of 5.9–6.3 [35], aminopropyl methacrylamide (APMA) hydrochloride was chosen as functional monomer to create a positively charged environment within the polymer network [36] favorable to viral vector loading. Vectors containing different genes were loaded in the hydrogels and then investigated regarding stability, controlled release, and transduction efficiency in human bone marrow derived mesenchymal stem cells (hMSCS), a potential cell population for cornea regenerative approaches [37,38], and in an ex vivo model of bovine cornea.

## 2. Materials and Methods

### 2.1. Materials

2-Hydroxyethyl methacrylate (2-HEMA) was supplied by Merck (Darmstadt, Germany); ethylene glycol dimethacrylate (EGDMA), Dulbecco’s Modified Eagle’s Medium (DMEM) and dichlorodimethylsilane were from Sigma-Aldrich (Steinheim, Germany); N-(3-aminopropyl) methacrylamide hydrochloride (APMA) was from Polysciences Inc. (Warrington, PA, USA); D(+)-sucrose (99.7%) and 2,2′-azo-bis(isobutyronitrile) (AIBN) from Acros (Geel, Belgium). The Cy3 Ab Labeling Kit was supplied from Amersham/GE Healthcare (Munich, Germany). The AAV Titration ELISA was from Progen (Heidelberg, Germany) and the IGF-1 ELISA kit (Insuline like Growth Factor 1) from R&D Systems (Nordenstadt, Germany); the Beta-Glo^®^ Assay System was from Promega (Mannheim, Germany); the β-gal staining kit and cell proliferation reagent WST-1 were obtained from Roche Applied Science (Mannheim, Germany). Vectastain ABC HRP kit (Peroxidase, Standard) and Biotynilated Dolichos Biflorus Agglutinin (DBA) were from Vector Laboratories (Burlingame, CA, USA). The antibody anti-IGF-I (AF-291-NA) was from R&D Systems (Nordenstadt, Germany). Ultra-pure water (resistivity > 18.2 MΩ·cm) was obtained by reverse osmosis (MilliQ^®^, Millipore Ibérica, Madrid, Spain) and water for cell culture was from Sigma-Aldrich (Steinheim, Germany). All other reagents were analytical grade.

### 2.2. Cell Culture

hMSCs were obtained of bone marrow aspirates (15 mL) from the distal femur of patients undergoing total knee arthroplasty (*n* = 3, age 65–72 years) [20,37]. The Ethics Committee of the Saarland Physicians Council approved the study. All patients provided informed consent before inclusion in the study. All procedures were in accordance with the Helsinki Declaration. hMSCs were isolated and expanded in culture using standard protocols. Cells (passage 1) were seeded in 48-well plates (5000 cells/well), maintained in DMEM, 10% FBS, 100 IU/mL penicillin G, 100 mg/mL streptomycin (growth medium), and incubated at 37 °C for 12 h before adding hydrogels. The 293 cell line, an adenovirus-transformed human embryonic kidney cell line, was used for rAAV packaging and maintained in growth medium (the same medium as hMSCs) [20,39].

### 2.3. Hydrogel Synthesis

Monomer mixtures were prepared with the composition shown in Table 1. Components were placed into glass vials and mixed at room temperature under magnetic stirring (400 rpm, 30 min). Monomer solutions were injected into molds constituted by two glass plates (10 × 10 cm) pretreated with dichlorodimethylsilane and separated by a silicone frame of 0.5 mm thickness. The molds were then placed in an oven at 50 °C for 12 h and then heated to 70 °C for 24 h more. After polymerization, the hydrogel sheets were immersed in boiling water (500 mL) for 15 min and then cut into discs (10 mm in diameter). These discs were dried at 70 °C for 3 h and stored in airtight plastic bags.

### 2.4. Hydrogel Characterization

The degree of swelling was determined, in triplicate, as the increase in weight of dried discs placed into vials containing sucrose (10%) aqueous solution. The experiments were carried out at 4 °C and also at room temperature (≈15–20 °C, after heating in autoclave at 121 °C, 21 min). At predetermined time intervals, the discs were removed from the medium, the surface wiped with filter paper and then the discs were weighed and returned to the corresponding vial. The degree of swelling was calculated as follows:

(1) $$Swelling\;degree\;\left(\%\right)=\frac{W_{t}-W_{0}}{W_{0}}x100$$

*W*_0_ and *W_t_* represent the weight of dried hydrogel and of wet hydrogel at time t, respectively.

The transmittance at 600 nm of fully hydrated discs in sucrose aqueous solution (10%) at room temperature was measured, in triplicate, in an Agilent 8453 spectrophotometer (Germany) in duplicate. Oxygen permeability of the hydrogels swollen in 0.9% NaCl at room temperature for 24 h was measured, in duplicate, using a Createch permeometer model 210T (Rehder Development Company, Castro Valley, CA, USA) fitted with a flat cell in a 100% RH chamber. Current intensity was recorded when stabilized (in the first 5 min on the flat cell) and the dark current was taken into account for the oxygen permeability calculations.

### 2.5. Plasmids and rAAV Vectors

The constructs were derived from pSSV9, an AAV-2 genomic clone [40,41]. rAAV-*lacZ* carries the *lacZ* gene encoding *Escherichia coli* β-galactosidase (β-gal), rAAV-RFP the *Discosoma* sp. red fluorescent protein (RFP) gene, and rAAV-hIGF-I a human insulin-like growth factor I (hIGF-I) cDNA (536 bp), all under the control of the cytomegalovirus immediate-early promoter [39,42,43,44]. The vectors were packaged as conventional (not self-complementary) vectors using a helper-free, two-plasmid transfection system in 293 cells with the packaging plasmid pXX2 and the adenovirus helper plasmid pXX6 as previously described [39,42,43,44]. The vector preparations were purified by dialysis and titrated by real-time PCR [39,42,43,44], averaging 10^10^ transgene copies/mL (≈1/500 functional recombinant viral particles).

### 2.6. Incorporation of rAAV Vectors to Hydrogels

Discs of hydrogel (Hc, H_1_, and H_2_, in triplicate) were moistened with water to facilitate the cut into pieces of 9 mm^2^ each (≈7 mg). Then, the pieces were dried at 60 °C for 3 h and transferred to vials containing sucrose aqueous solution (10%; 3 mL, viral particles preservation medium) for sterilization in autoclave (121 °C, 20 min). After sterilization, the vials were stored at 4 °C until use (≈12 h).

Loading assay was carried out, in triplicate, in 96-well plates adding 100 µL of rAAV-*lacZ* dispersed in sucrose aqueous solution (10%, containing 1.6 × 10^8^ capsids) to each hydrogel piece. The plate was incubated at 4 °C in an orbital shaker (60 osc/min) for 24 h. Then, the loading medium was taken and stored at −20 °C. The amounts of rAAV-*lacZ* in the loading medium were quantified by AAV2 titration ELISA (Progen Biotechnik GmbH; Heidelberg, Germany). The amounts of vector loaded by the hydrogels were calculated as follows:

(2) $${rAAV}_{t}=\frac{rAAV_{0}-rAAV_{f}}{W_{dh}}$$

where *rAAV*_0_ and *rAAV_f_* represent the amounts of rAAV capsids in the medium at the beginning of the assay and after 24 h, respectively, and *W_dh_* is the weight of dry hydrogel. In addition, partition coefficient (K_N/W_) was calculated as follows [45]:

(3) $$rAAV_{t}=\frac{Vs+K_{N/W}x\;Vp}{Wp}xC_{0}$$

where *Vs* is the volume of water sorbed by the hydrogel, *Vp* the volume of dried polymer, *Wp* the dried hydrogel weight, and *C*_0_ the concentration of viral vector in the loading solution. Finally, the functional monomer factor (FMF) [31] was calculated as the ratio between amounts of rAAV loaded by functionalized hydrogels divided by the amount of rAAV loaded by the HEMA hydrogel without APMA.

### 2.7. Loading of Cy3-Labelled-rAAV

Two discs of each hydrogel (H_c_, H_1_, and H_2_) were transferred to vials containing sucrose aqueous solution (10%; 3 mL) for sterilization in autoclave (121 °C, 20 min). After sterilization, the vials were stored at 4 °C until use (≈12 h). Then, discs were individually placed in 48-well plates with rAAV-*lacZ* vectors dispersed in sucrose aqueous solution (10%, 250 μL) and previously labeled using the Cy3 Ab Labeling Kit according to the manufacturer’s recommendations and as previously described [20,46]. The plate was incubated at 4 °C for 24 h under orbital shaking (60 rpm). Effective loading of rAAV was monitored by live fluorescent microscopy with a rhodamine filter set (Olympus CKX41, Hamburg, Germany).

### 2.8. Release of rAAV from Hydrogels

Hydrogels (Hc, H_1_, and H_2_) loaded with rAAV-*lacZ* (as in Section 2.6) were transferred to 48-well plates, immersed in 250 µL of DMEM and kept at 37 °C under orbital shaking (200 rpm). Aliquots (250 μL) of culture medium were collected and immediately frozen at −20 °C at pre-established time points (6 h, 1, 7, and 14 days). Each well was replenished with the same volume of medium. Amounts of rAAV-*lacZ* released were quantified using AAV2 titration ELISA [20,47]. The assay was carried out in triplicate. Total amounts of rAAV released were expressed as the amount of released viral particles accumulated in the medium since the beginning of the assay (*R*_0_) [47].

(4) $$rAAV\;released=\sum R_{0\to t}$$

Hydrogels loaded with Cy3-rAAV-*lacZ* were also immersed in DMEM (250 μL) at 37 °C under orbital shaking (200 rpm) and the amounts of rAAV remnant inside hydrogels were monitored by fluorescence at 1, 7, and 14 days.

### 2.9. Cell Viability

Loaded and non-loaded pieces of hydrogel (9 mm^2^; Hc, H_1_, and H_2_) were placed, in triplicate, in 48-well plates on hMSCs monolayer at 37 °C, 5% CO_2_, and 95% RH. Sucrose aqueous solution (10%, 25 μL) was used as negative control and a dispersion of rAAV-*lacZ* in sucrose aqueous solutions (10%, 25 μL) as positive control. Cell viability was evaluated using WST-1 Cell Proliferation Reagent (Roche, Switzerland). Both loaded and non-loaded hydrogels were removed from the wells at pre-established periods of time, and the cell proliferation assay was carried out following the instructions from the manufacturer. Absorbance was read at 450 nm on a Tecan GENios Microplate Reader (Männedorf, Switzerland). The experiments were carried out in duplicate and cell viability (%) was calculated using the following equation:

(5) $$Cell\;viability\;\left(\%\right)=\frac{Abs_{sample}}{Abs_{negative\;control}}x100$$

### 2.10. Transgene Expression of hMSC Monolayers Using rAAV-Loaded Hydrogels

Pieces of hydrogel (9 mm^2^; H_c_, H_1_, and H_2_) were loaded with rAAV-*lacZ*, rAAV-RFP, or rAAV-hIGF-I using the conditions described in Section 2.6. Loaded-hydrogels were placed immediately into 48-well plates containing hMSCs monolayers (5000 cells/well for rAAV-*lacZ* and rAAV-RFP or 10,000 cells/well for rAAV-hIGF-I) and 250 μL of growth medium. Cultures were maintained at 37 °C, 5% CO_2_, and 95% RH for 14 days.

Expression of the transgene (*lacZ*) was determined in the cell culture after 1, 7, and 14 days by X-Gal staining (using a β-gal staining kit, one replicate) and the Beta-Glo^®^ Assay System (in duplicate). Quantitative measurements were performed on a Tecan GENios Microplate Reader (Männedorf, Switzerland) and expressed as relative luminescence units (RLU) standardized by number of cells seeded [48]. RFP was monitored overtime by live fluorescence imaging with an Olympus CXK41 inverted microscope (Tokyo, Japan).

Expression of IGF-I was quantified using an ELISA kit. hMSCs monolayers, where hydrogels were placed, were washed twice with DMEM (serum-free). Then, the medium was replaced by serum-free medium 24 h before collection of culture medium supernatants for quantification of IGF-I expression. hMSCs with serum-free medium were incubated at 37 °C, 5% CO_2_, and 95% RH. Supernatants were collected at the denoted time points (1, 7, and 14 days) and centrifuged to remove debris. Absorbance at 450 nm was measured using a GENios spectrophotometer/fluorometer (Tecan, Crailsheim, Germany).

### 2.11. Metabolic Activity of hMSCs Monolayers upon rAAV-hIGF-I Delivery from Hydrogels

Hydrogels (9 mm^2^, H_c_ and H_2_) loaded with rAAV-hIGF-I were placed, in triplicate, on hMSCs monolayers and proliferation was measured using the Cell Proliferation Reagent WST-1 at days 1, 7, and 14 [39]. Control conditions included cells maintained in the presence or absence of free vector and cells cultured with non-loaded hydrogel pieces in duplicate.

### 2.12. Immunohistochemical Analysis

Monolayer cultures of hMSCs in contact with hydrogels (9 mm^2^, H_c_ and H_2_) were fixed in 4% formalin and immunohistochemistry was performed at 1, 7, and 14 days after exposure, using anti-IGF-I-primary antibody, anti-goat IgG secondary antibody, biotinylated secondary antibodies, and the ABC method with diaminobenzidine as the chromogen. Cells maintained in the presence or absence of free vector and cells cultured with non-loaded hydrogel pieces in duplicate were used as controls. Monolayer pictures were recorded with an Olympus CXK41 inverted microscope (Tokyo, Japan).

### 2.13. Cornea Transduction Using rAAV-lacZ Loaded-Hydrogels

Fresh bovine eyeballs, immediately enucleated after animal slaughter, were collected from the local slaughterhouse and immersed in PBS placed in an ice bath. Cornea was excised within the same day and cut into samples of 9 mm^2^. Then, samples of cornea were incubated in DMEM (150 µL, 10% FBS, penicillin/streptomycin 100 IU/mL and 100 μg/mL, respectively) inside 96-well plates with corneal epithelium upwards. rAAV-*lacZ* loaded-hydrogels (Hc and H_2_; loaded as in Section 2.6) were deposited over the cornea and incubated for 7 days replacing the medium every 2 days. Free rAAV-*lacZ* dispersed in sucrose aqueous solution (25 µL, 10%) and sucrose aqueous solution (25 µL, 10%) were used as positive and negative controls, respectively. Samples were assayed in duplicate in two independent experiments. After incubation period ending, expression of the transgene (*lacZ*) was determined in the cornea by X-Gal staining (using a β-gal staining kit).

### 2.14. Statistical Analysis

Loading and release assays and transgene expression tests were performed in triplicate and the other conditions in duplicate in two independent experiments. Data were expressed as mean and standard deviation (SD). Statistical analysis was performed using Statgraphics Centurion version XVI.II by simple ANOVA and multiple range test with *p* ≤ 0.05 considered statistically significant.

## 3. Results

### 3.1. Hydrogel Synthesis and Characterization

**Figure 2:**
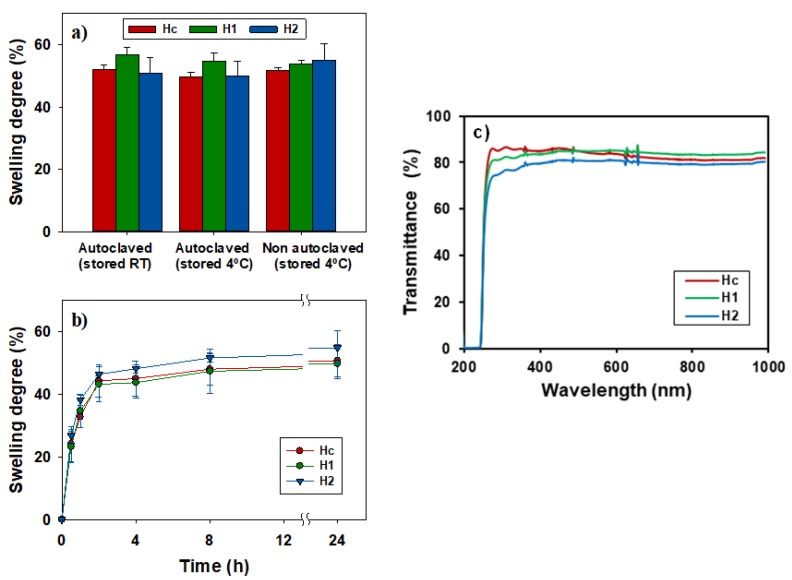
(**a**) Final swelling degree of H_c_, H_1_, and H_2_ discs stored at room temperature and at 4 °C for 24 h after autoclaving (121 °C, 21 min), and stored 24 h at 4 °C with no previous sterilization process; all in sucrose 10% aqueous medium; (**b**) evolution of swelling degree of H_c_, H_1_, and H_2_ discs in sucrose 10% at 4 °C; and (**c**) light transmittance profiles in the UV/visible range of H_c_, H_1_, and H_2_ hydrogel discs swollen in sucrose 10% aqueous solution.

The three sets of hydrogels H_c_, H_1_, and H_2_ prepared containing 0, 40, and 80 mM of APMA, respectively, showed a similar degree of swelling in sucrose 10% aqueous medium at 4 °C (H_c_: 50.8 (s.d. 5.1%); H_1_: 49.8 (s.d. 4.8)%; H_2_: 59.9 (s.d. 5.3)%, and at room temperature (≈15–20 °C) after sterilization (121 °C, 20 min). Swelling behavior of autoclaved discs was also similar after 24 h storage at 4 °C; H_c_: 52.0 (s.d. 1.5)%; H_1_: 49.7 (s.d. 1.4)%; and H_2_: 51.6 (s.d. 0.8)% (Figure 2a,b). Regarding light transmission, discs functionalized with APMA (40 and 80 mM) presented transmittance profiles above 80% in the 400–800 nm range. The transmittance decreased dramatically in the UV range allowing for eye protection against this radiation (Figure 2c). Oxygen permeability was about 14 barrer for the three sets of hydrogels.

### 3.2. Loading and Release of rAAV Vectors

Before loading, hydrogels were sterilized by steam heating (autoclave, 121 °C for 20 min) immersed in sucrose 10%, and then stored at 4 °C until use (≈12 h). The hydrogels were transferred to the rAAV dispersion and kept at 4 °C to maintain viral vector titer, for 24 h under oscillatory movement (60 osc/min, orbital shaker).

**Figure 3:**
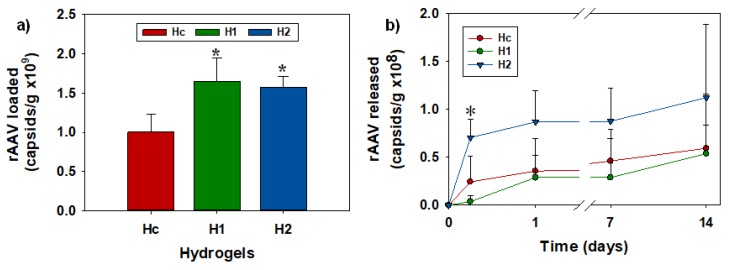
(**a**) Capsids of recombinant adeno-associated virus (rAAV)-*lacZ* loaded per gram of hydrogel (H_c_: control; H_1_: 40 mM aminopropyl methacrylamide (APMA); H_2_: 80 mM APMA) after 24 h of incubation in 100 μL of viral vector dispersion (1.6 × 10^8^ capsids) in sucrose 10% aqueous solution at 4 °C and 60 osc/min in 96-well plates. * Significant differences regarding hydrogel control, H_c_ (*p* < 0.05). (**b**) Release profiles of rAAV-*lacZ* from hydrogels in Dulbecco’s Modified Eagle’s Medium (DMEM) (0.25 mL) at 37 °C and 200 rpm. * Significant differences compared to hydrogel control (H_c_) and H_1_ (*p* < 0.05).

Control hydrogel discs (H_c_) loaded 43.0 (s.d. 9.6)% of rAAV-*lacZ* capsids (1.0 ± 0.2 × 10^9^ capsids/g of hydrogel) from rAAV loading solution. Functionalization with low proportions of APMA was enough to increase the loading of rAAV-*lacZ* up to 70.7 (s.d. 12.9)% for H_1_ and 67.4 (s.d. 6.0)% for H_2_ (1.65 ± 0.3 and 1.57 ± 0.14 × 10^9^ capsids/g for H_1_ and H_2_, respectively). H_1_ and H_2_ loading data showed significant differences compared with H_c_ (Figure 3a, * *p* < 0.05). The network/water partition coefficients (*K_N/W_*), which indicate the affinity of the viral particles for the networks, were as follows: H_c_: 5.78 (s.d. 1.85), H_1_: 9.27 (s.d. 2.12), and H_2_: 8.81 (s.d. 2.76). Hydrogels were also loaded with Cy3-rAAV-*lacZ*, and the fluorescence measurements confirmed the vector uptake (Figure S1, Supporting Information).

Release tests were carried out in DMEM at 37 °C to mimic cell culture conditions [48]. Adjacent wells were also filled with the same medium and the plate sealed with parafilm to minimize the evaporation. All hydrogels showed controlled release of viral vectors (Figure 3b). The hydrogel with APMA 80 mM (H_2_) released higher amounts of capsids for 14 days; the amounts released at 6 h were significantly different (*p* < 0.05) than those released from control (H_c_) and 40 mM APMA hydrogel (H_1_). The amount of vector released was roughly 10% of the amount previously loaded, which may be due to a strong retention of the capsids in the hydrogels and to the instability of the vector at the assay conditions (37 °C) [49].

### 3.3. Cytocompatibility

**Figure 4:**
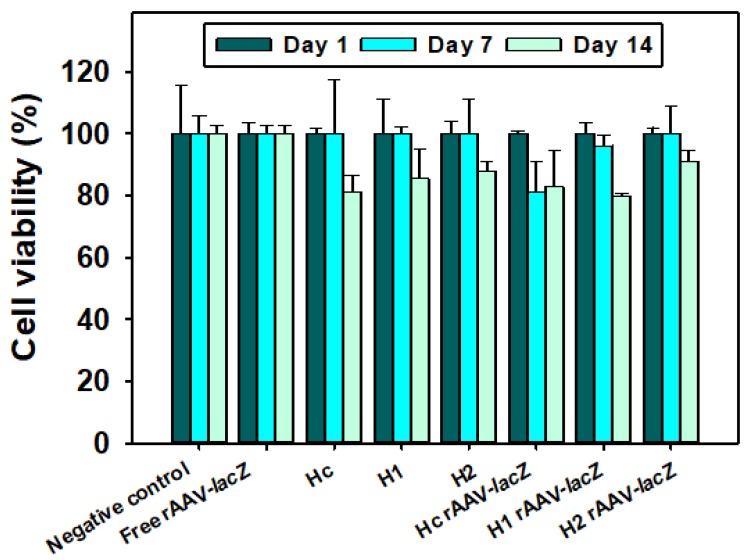
Cell viability of human bone marrow derived mesenchymal stem cells (hMSCs) monolayers incubated with free viral vectors, rAAV-lacZ-loaded hydrogels and non-loaded hydrogels for 14 days at 37 °C, 5% CO_2_, and 95% RH.

Hydrogels H_c_, H_1_, and H_2_ loaded with rAAV-lacZ were tested on hMSCs monolayer in DMEM using WST-1 Cell Proliferation Reagent, and compared with non-loaded hydrogels and free rAAV-lacZ in sucrose 10% aqueous solution. Cells cultured with sucrose 10% aqueous solution (25 μL) were used as negative control. Cytocompatibility was close to 100% at day 7 and still above 80% at day 14 for both rAAV-lacZ loaded and non-loaded hydrogels (Figure 4), which suggests that the viral vector-APMA-functionalized hydrogel combination product may be highly biocompatible.

### 3.4. Gene Transfer Efficiency via rAAV Controlled Release from Hydrogels

**Figure 5:**
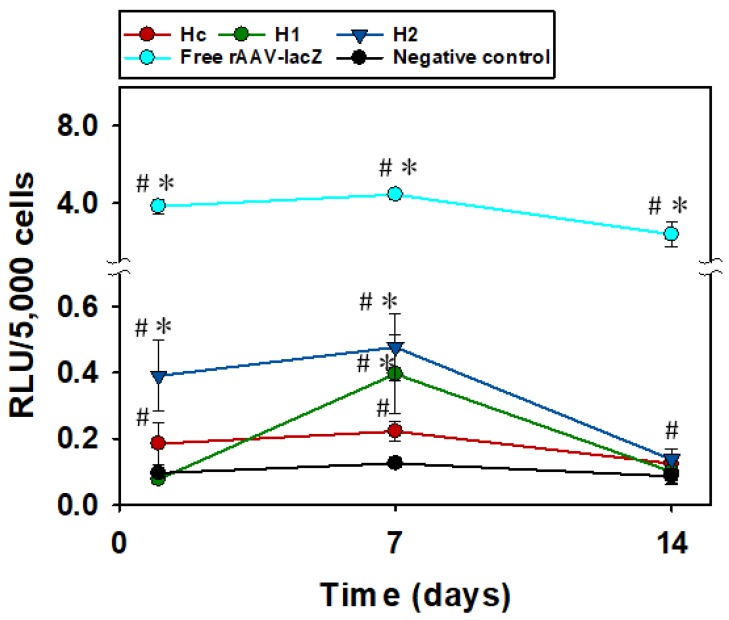
Expression of transgene *lacZ* by hMSCs monolayer after being in contact with rAAV-*lacZ* loaded hydrogels (Hc, H_1_, and H_2_) and free rAAV-*lacZ* for 1, 7, and 14 days, quantified through Beta-Glo^®^ Assay System and standardized by number of cells seeded. (#) Significant differences regarding negative control and (*) significant differences regarding hydrogel control (Hc); both *p* < 0.05. It should be noted that the concentration of rAAV-*lacZ* was one order of magnitude higher in the case of free rAAV-*lacZ* compared to the amount of capsids that the hydrogels could release.

The ability of rAAV-loaded hydrogels to effectively transduce hMSCs monolayers was tested by using reporter genes for β-galactosidase activity (lacZ) and for red fluorescent protein (RFP). Quantification of rAAV-lacZ expression through luminescence (Figure 5) showed that hydrogels functionalized with 80 mM APMA (H_2_) can control the transduction of rAAV for 7 days (* *p* < 0.05), and at day 7 all hydrogels with and without APMA were able to transduce cells (# *p* < 0.05, compared with the negative control). The expression of the transgene lacZ was higher for H_2_ in the first hours in good agreement with the release test results. Then, hydrogels and free vector rAAV-lacZ showed transduction decrease between 7 and 14 days, which suggests the cells remained permissive to rAAV-mediated gene transfer over time but expressed the transgene at lower levels. Moreover, amounts of free rAAV-lacZ used were 10 times higher than those loaded within hydrogel pieces, which explains the difference in transduction levels. Pictures from X-Gal staining (Figure S2, Supporting Information) of β-galactosidase agree well with results obtained in quantitative Beta-Glo^®^ assay. Fluorescence images of RFP (Figure S3, Supporting Information) also confirmed the ability of hydrogels to deliver active rAAV to transduce cells.

### 3.5. rAAV-hIGF-I Delivery to hMSCs

**Figure 6:**
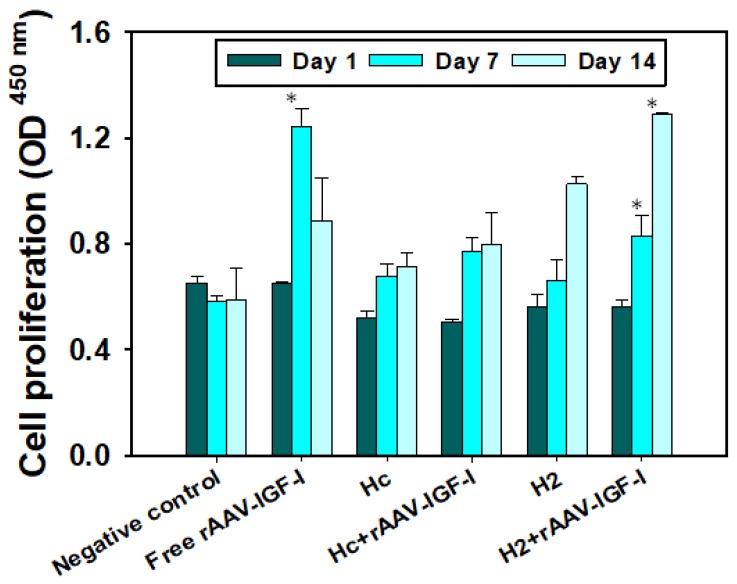
Cell proliferation (hMSCs) after direct contact with hydrogels with and without rAAV-hIGF-I for 1, 7, and 14 days. * *p* < 0.05 regarding the controls with no vector.

Assessment of hydrogels as platforms for gene therapy to induce a therapeutic response was performed using insulin growth factor-I (IGF-I) as therapeutic gene. IGF-I was selected due to the feasibility of quantification of its effect by means of cell proliferation tests and its role in the maintenance of corneal epithelial homeostasis [50]. hMSCs monolayers were exposed to hydrogels with and without rAAV-hIGF-I and incubated at 37 °C, 5% CO_2_, and 95% RH. Cells in contact with H_2_ hydrogels loaded with rAAV-hIGF-I showed increased proliferation up to 14 days, while free rAAV-hIGF-I increased cell proliferation at day 7 and then decreased at day 14. Diminished cell proliferation at day 14 was due to lower levels of transduction provided by the free vector (as shown in the gene transfer efficiency assay; Figure 5). Differently, H_2_ discs sustained the delivery of the therapeutic genes and triggered therapeutic response for 14 days, showing increased cell proliferation (Figure 6). Immunohistochemical staining of hMSCs confirmed that IGF-I expression was higher when cells were exposed to hydrogels loaded with rAAV-hIGF-I regarding the control hydrogels without vector (Figure S4, Supporting Information).

### 3.6. Cornea Transduction Using rAAV-lacZ Loaded-Hydrogels

**Figure 7:**
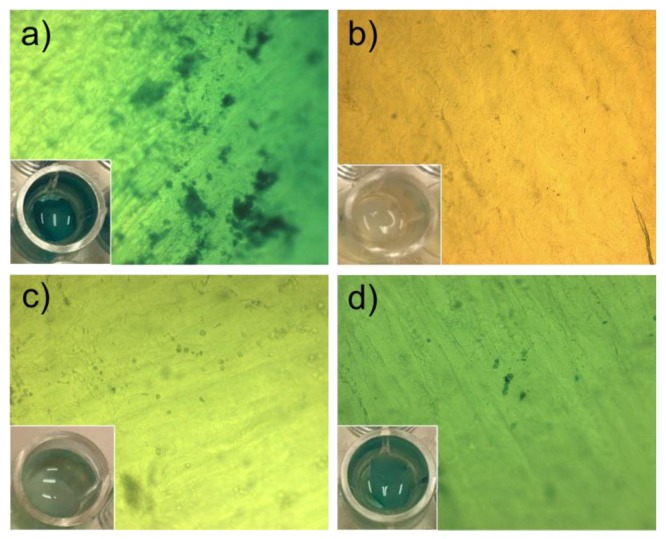
Pictures of X-Gal stained bovine corneas (×10 magnification) after 7 days in direct contact with (**a**) free rAAV-*lacZ*, (**b**) control sucrose 10% aqueous solution, (**c**) rAAV-*lacZ* loaded-Hc hydrogel (0 mM APMA), and (**d**) rAAV-*lacZ* loaded-H_2_ hydrogel (80 mM APMA). Pictures on the bottom left represent macroscopic appearance of corneas within a 96-well plate. It should be noted that the concentration of rAAV-*lacZ* was one order of magnitude higher in the case of free rAAV-*lacZ* compared to the amount of capsids that the hydrogels could release.

Hydrogels loaded with rAAV-lacZ were placed in direct contact with bovine cornea explants and evaluated regarding rAAV-lacZ transduction (Figure 7). Since rAAV-lacZ released from H_2_ showed the maximum transduction in hMSCs at day 7 (Figure 5), the corneal assay was also prolonged for 7 days. The corneas were placed with the epithelium layer upwards to keep a direct contact with the hydrogel pieces (H_c_ and H_2_) mimicking CL wearing. Exposition to the free rAAV-*lacZ* vectors was also monitored (Figure 7a). The X-Gal staining revealed colored tissue for both H_c_ and H_2_ hydrogels loaded with rAAV-lacZ, although H_2_ hydrogels led to more intense blue color (Figure 7c,d). False positive colorations were discarded applying the same staining protocol to non-treated corneas (Figure 7b).

## 4. Discussion

### 4.1. Hydrogel Synthesis and Characterization

APMA hydrochloride was chosen as functional monomer to endow the polymeric network with positively charged moieties [36] which may create a favorable environment for viral vector incorporation and retention due to rAAV vector surface negative charges [34,35]. APMA readily dissolved until 80 mM in HEMA, but higher concentrations would require addition of cosolvents. After polymerization, hydrogels were washed in boiling water to remove remaining unreacted monomers and facilitate the cut into discs. Finally, the discs were dried and stored at room temperature in airtight plastic bags.

Before the assays, the discs were cut into pieces of approximately 9 mm^2^, dried and weighed (≈7 mg), immersed in sucrose 10% aqueous solution (5 mL) and sterilized. Sucrose 10% medium was used because it is an isotonic and common maintenance medium for viral vectors. Incorporation of APMA as functional comonomer did not cause significant changes in the swelling degree, light transparency, and oxygen permeability of the hydrogels, with values in the range of those of commercially available hydrogel CLs.

### 4.2. rAAV Loading and Release

Temperature, composition of the medium, and time may play an important role in viral capsid degradation [49]. Thus, before loading, hydrogels were immersed in sucrose 10% aq. solution, autoclaved and stored at 4 °C. Viral capsids have been shown to be stable for several days in sucrose 10% medium at 4 °C [49] and thus these conditions were used to carry out the loading into the hydrogels. It is noticeable that although the proportion of APMA (≈1 mol%) was quite low compared to that of the structural monomer HEMA, the presence of APMA caused a remarkable increase in the capability of the hydrogels to host the viral vectors. Assuming complete polymerization, the number of cationic moieties available in H_1_ and H_2_ hydrogels would be 2.4 × 10^19^ and 4.8 × 10^19^ per gram, respectively. Multiple-point interactions with the anionic capsids cannot be discarded [51]. Therefore, the amount of capsids that could be loaded through electrostatic binding could be higher if the loading would be carried out in a more concentrated capsids medium. Interestingly, the K_N/W_ values indicate that the capsids also exhibit affinity for the control networks, H_c_, which means that hydrophobic interactions and hydrogen bonding with the HEMA-EGDMA network also play a role in the loading [51]. Nevertheless, the electrostatic interactions enhanced the affinity two-fold. Indeed, APMA-functionalized hydrogels presented functional monomer factor (FMF) values well above 1 (FMF of H_1_: 1.64 and H_2_: 1.57) which is a clear indication of the positive role that APMA plays in the loading of the viral vectors.

The functionalized hydrogels prepared with the highest content in APMA were the ones that released more viral vectors in the first 24 h. Although exposition at 37 °C may be responsible for only 10% viral vectors previously loaded being detected in the release study [49], the inner environment of the hydrogel with the highest content in functional groups (i.e., H_2_: 80 mM APMA) seems to stabilize rAAV-*lacZ*, increasing the amount of vector available to transduce cells. Notwithstanding above, the amount of viral vectors released in the first hours could be already useful for corneal treatment, since 10^7^–10^10^ gene copies have been shown efficient for transfection at ocular level and in other tissues [48,52].

### 4.3. Gene Transfer Efficiency

Once the designed hydrogels demonstrated high compatibility with hMSCs (according to ISO 10993-5, cell viability values above 70% indicate absence of toxicity), the next step was to verify that they can deliver the viral vectors for efficient cell transduction using reporter genes for both β-galactosidase activity (*lacZ*) and red fluorescent protein (RFP). Once again, the hydrogel with the highest content in functional groups (H_2_: 80 mM APMA) provided the highest transgene expression in the first hours in good agreement with the release test results. Moreover, the hydrogels sustained the transduction for several days. Thus, although wearing of HEMA-based CLs is limited to one or few days depending on the oxygen permeability, changes in the composition of the structural monomers while maintaining the functional APMA may open the possibility of developing extended wear CLs that can fully develop prolonged transduction on cornea surface. It is interesting to note that compared to the direct application of the free viral vector, using the APMA-functionalized hydrogels as platforms for the sustained release allows more progressive and more prolonged therapeutic effects, as confirmed with the IGF-I therapeutic gene. Finally, as a proof of concept, the hydrogels loaded with rAAV-*lacZ* were challenged against cornea transduction mimicking the CL wearing conditions. The intense color caused by the APMA-functionalized hydrogels confirmed the success of the approach, opening novel ways of addressing ocular gene therapy overcoming the limitations of both eye drops (short permanence time) and intraocular injections (patient discomfort and risk of tissue damage). From a translational point of view, this approach will require a precise regulation by using tissue-specific, inducible or tissue-responsive promoters [53,54,55] rather than the ubiquitous CMV-promoter.

## 5. Conclusions

HEMA hydrogels functionalized with APMA preserved appropriate characteristics to be placed on the cornea surface regarding wettability, transparency, and oxygen permeability. Furthermore, functionalization with APMA increased rAAV vector loading capability and endowed hydrogels with ability to release more viral copies in a controlled manner in a time frame compatible with the wearing of CLs. Hydrogels functionalized with 80 mM APMA demonstrated increased transduction of reporter genes for both β-galactosidase activity (*lacZ*) and for red fluorescent protein (RFP) in a potential reparative population of hMSCs, compared with no functionalized ones. Relevant from a clinical perspective, overexpression of hIGF-I, a growth factor involved in normal cornea homeostasis, via rAAV using functionalized hydrogels significantly increased cell proliferation, proving the ability of the designed formulation to better control rAAV release and gene transduction for 14 days. This first attempt to develop hydrogels able to act both as rAAV delivery platforms for corneal gene therapy and as contact lenses for correction of refractive errors may open novel ways of addressing eye diseases.

## Figures and Tables

**Table 1:** Composition of monomers mixtures used to prepare the hydrogel contact lenses.

Hydrogel	HEMA (mL)	EGDMA (µL/mM)	AIBN (mg/mM)	APMA (mg/mM)
**H_C_**	3	45.20/80	4.93/10	0/0
**H_1_**	3	45.20/80	4.93/10	21.40/40
**H_2_**	3	45.20/80	4.93/10	42.80/80

## Supplementary Materials

The following are available online at https://www.mdpi.com/1999-4923/12/4/335/s1. Figure S1: Fluorescence pictures of Cy3-rAAV-*lacZ*-loaded hydrogels (day 0) and hydrogels after 1, 7, and 14 days of release where controls are non-laden discs. Figure S2: Pictures from X-Gal staining of hMSCs monolayer (×10 magnification) after 1, 7, and 14 days in direct contact with hydrogels, control medium (sucrose 10%), and free rAAV-*lacZ*. Figure S3: Fluorescence images of RFP (×10 magnification) after 1, 7, and 14 days after direct contact of hMSCs with hydrogels and free rAAV-RFP. Figure S4: Pictures of immunohistochemical staining of hMSCs monolayer after 1, 7, and 14 days with H_c_ and H_2_ hydrogels with and without rAAV-hIGF-I. Free rAAV-hIGF-I was used as positive control, and sucrose 10% as negative control.
