# Supplementary material for: Controlled Release of rAAV Vectors from APMA-Functionalized Contact Lenses for Corneal Gene Therapy
Source: Pharmaceutics. 2020 Apr 9;12(4):335. doi: 10.3390/pharmaceutics12040335 (PMC7238179; doi:10.3390/pharmaceutics12040335)
Supplement: Supplementary file 1 [file pharmaceutics-12-00335-s001.pdf]

SUPPORTING INFORMATION

Controlled release of rAAV vectors from APMA-functionalized contact lenses for corneal gene therapy

Fernando Alvarez-Rivera [1,§], Ana Rey-Rico [2,§], Jagadeesh K Venkatesan [3], Luis Diaz-Gomez [1], Magali Cucchiarini [3], Angel Concheiro [1], Carmen Alvarez-Lorenzo [1,*]

[1] Departamento de Farmacología, Farmacia y Tecnología Farmacéutica, I+DFarma, Facultad de Farmacia and Health Research Institute of Santiago de Compostela (IDIS), Universidade de Santiago de Compostela, 15782 Santiago de Compostela, Spain; fer.alvarez.rivera@gmail.com; luis.diaz.gomez@usc.es; angel.concheiro@usc.es; carmen.alvarez.lorenzo@usc.es

[2] Centro de Investigacións Científicas Avanzadas (CICA), Universidade da Coruña, Campus de A Coruña, 15071 A Coruña, Spain; ana.rey.rico@udc.es

[3] Center of Experimental Orthopaedics, Saarland University Medical Center, Homburg, Germany; jegadish.venki@gmail.com; mmcucchiarini@hotmail.com

[§]Equal contribution. Shared first authorship.

* Correspondence: carmen.alvarez.lorenzo@usc.es; Tel.: +34 881815239 (C.A.L.)

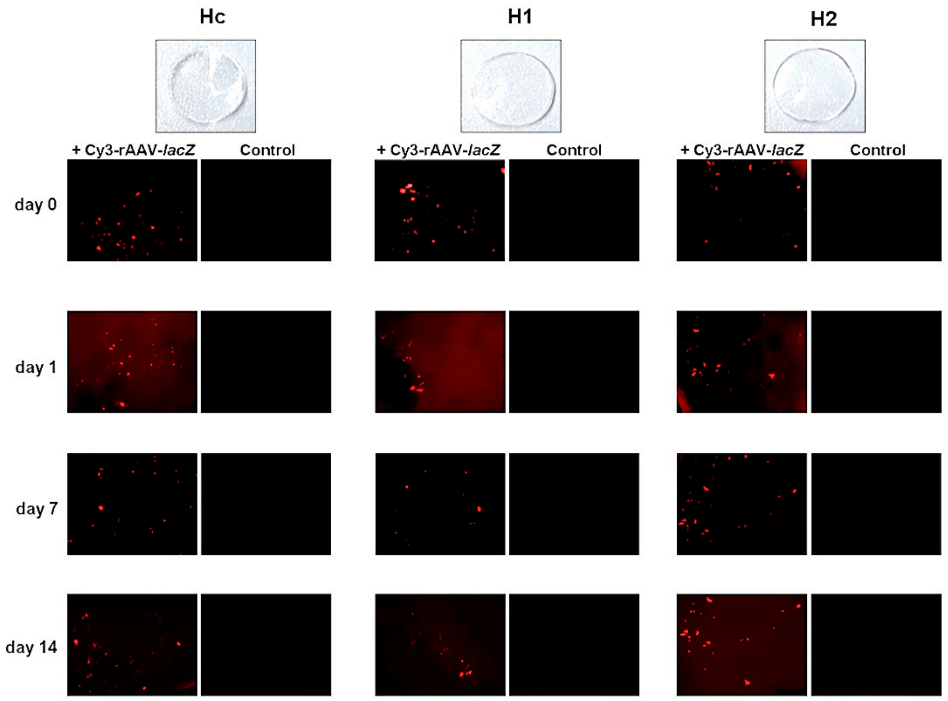

Figure S1: Fluorescence pictures of Cy3-rAAV-*lacZ*-loaded hydrogels (day 0) and hydrogels after 1, 7, and 14 days of release where controls are non-laden discs. Hydrogels appearance (10 mm in diameter) on the top of the figure.

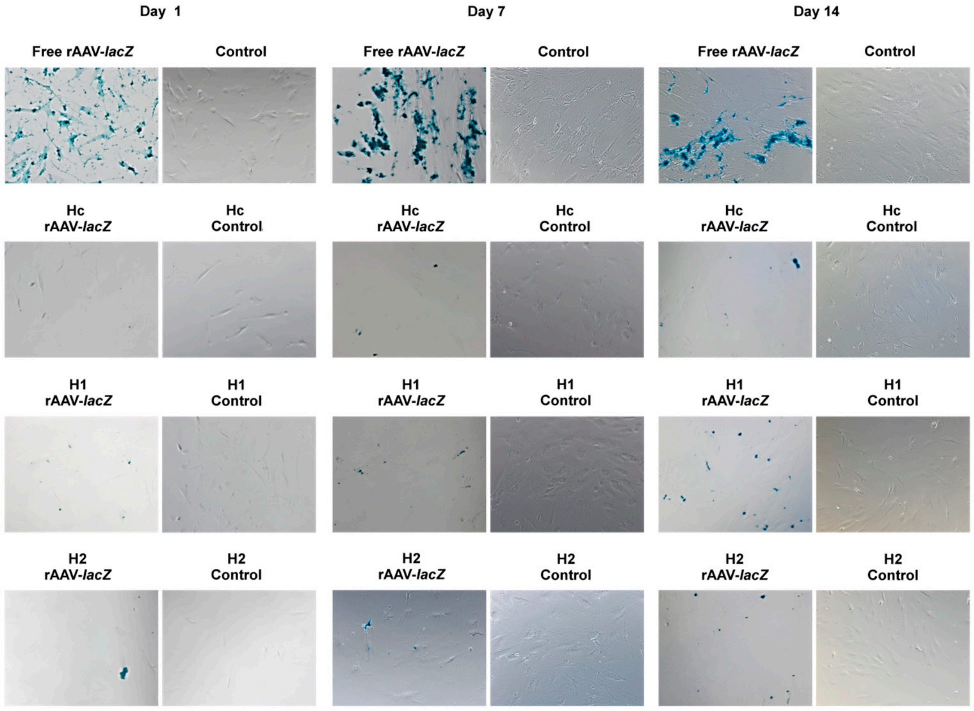

Figure S2: Pictures from X-Gal staining of hMSCs monolayer (x10 magnification) after 1, 7, and 14 days in direct contact with hydrogels, control medium (sucrose 10%), and free rAAV-*lacZ*.

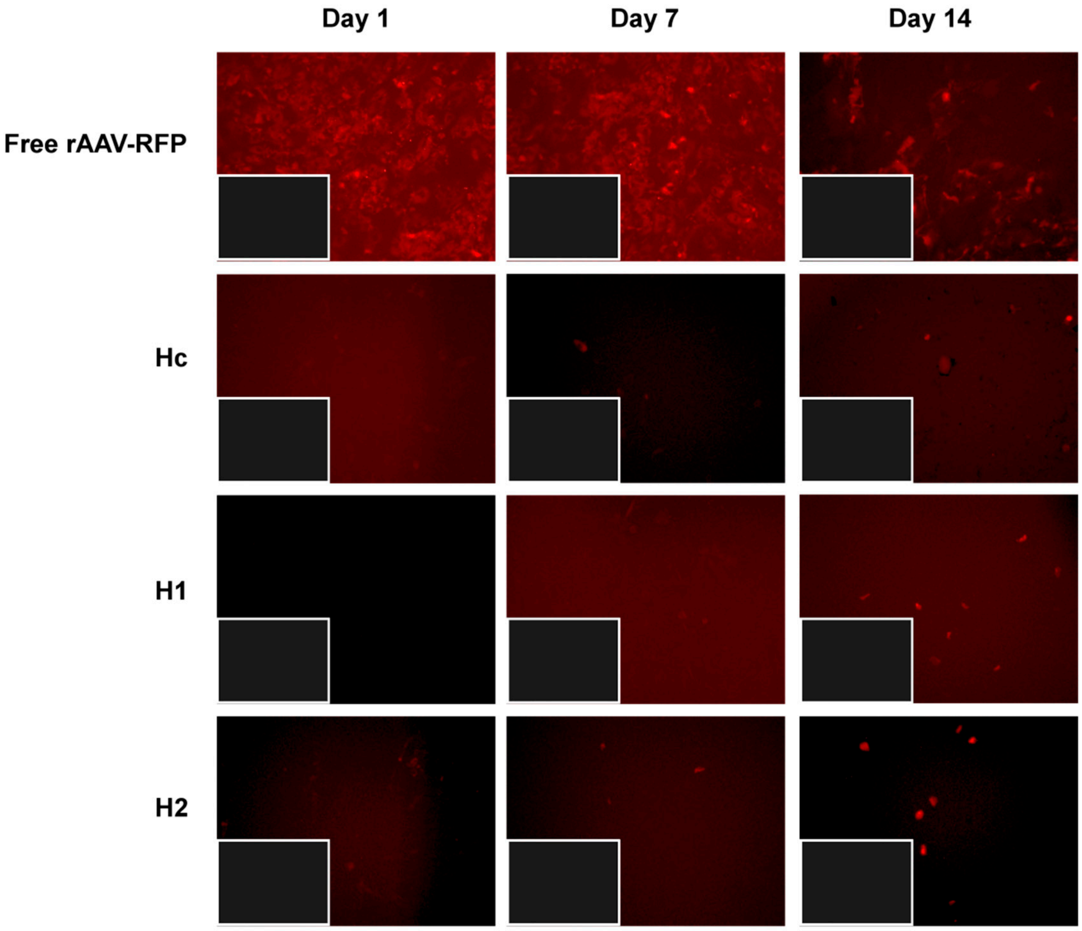

Figure S3: Fluorescence images of RFP (x 10 magnification) after 1, 7, and 14 days after direct contact of hMSCs with hydrogels and free rAAV-RFP. Small squares on bottom left represent negative control (sucrose 10% or Hc, H_1, and H_2 without rAAV-RFP) of each sample.

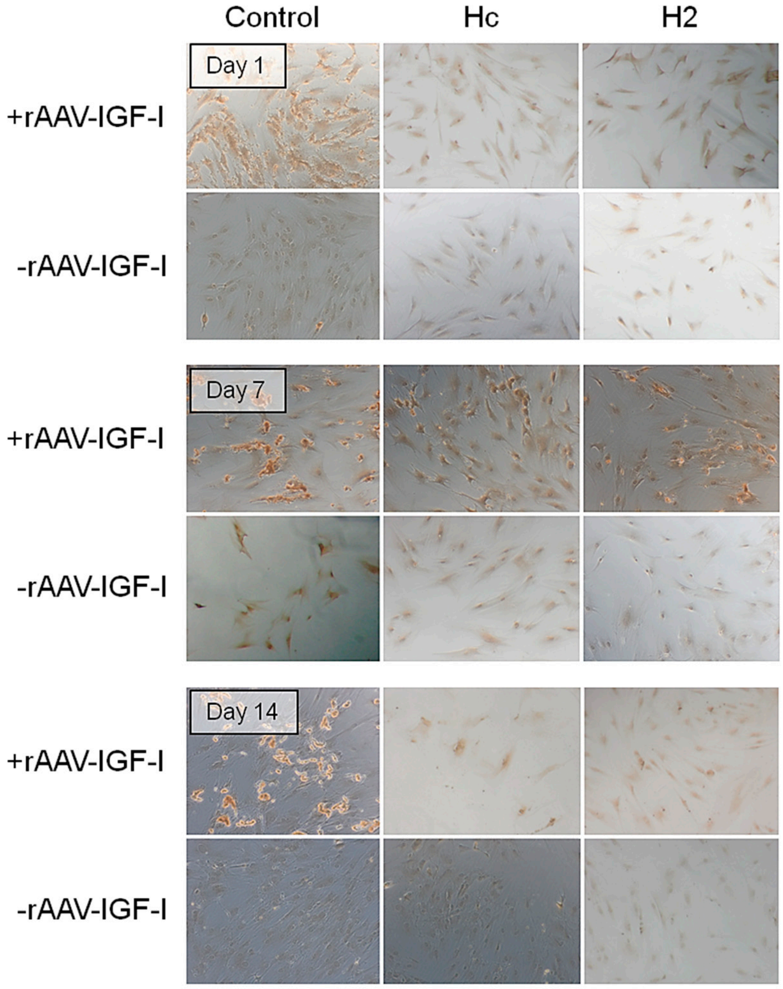

Figure S4: Pictures of immunohistochemical staining of hMSCs monolayer after 1, 7, and 14 days with Hc and H_2 hydrogels with and without rAAV-hIGF-I. Free rAAV-hIGF-I was used as positive control, and sucrose 10% as negative control.
